# Saturated Fatty Acid Activates T Cell Inflammation Through a Nicotinamide Nucleotide Transhydrogenase (NNT)-Dependent Mechanism

**DOI:** 10.3390/biom9020079

**Published:** 2019-02-25

**Authors:** Grace McCambridge, Madhur Agrawal, Alanna Keady, Philip A. Kern, Hatice Hasturk, Barbara S. Nikolajczyk, Leena P. Bharath

**Affiliations:** 1 Department of Nutrition and Public Health, Merrimack College, North Andover, MA 01845, USA; mccambridgeg@merrimack.edu (G.M.); keadya@merrimack.edu (A.K.); 2 Department of Pharmacology and Nutritional Sciences, University of Kentucky, Lexington, KY 40506, USA; m.agrawal@uky.edu; 3 Department of Medicine, University of Kentucky, Lexington, KY 40506, USA; pake222@uky.edu; 4 Barnstable Brown Diabetes and Obesity Center, University of Kentucky, Lexington, KY 40506, USA; 5 Forsyth Institute, Cambridge, MA 02142, USA; hhasturk@forsyth.org

**Keywords:** mitochondria, nicotinamide nucleotide transhydrogenase, oleate, palmitate, peripheral blood mononuclear cells, reactive oxygen species, Th17 cytokines

## Abstract

Circulating fatty acids (FAs) increase with obesity and can drive mitochondrial damage and inflammation. Nicotinamide nucleotide transhydrogenase (NNT) is a mitochondrial protein that positively regulates nicotinamide adenine dinucleotide phosphate (NADPH), a key mediator of energy transduction and redox homeostasis. The role that NNT-regulated bioenergetics play in the inflammatory response of immune cells in obesity is untested. Our objective was to determine how free fatty acids (FFAs) regulate inflammation through impacts on mitochondria and redox homeostasis of peripheral blood mononuclear cells (PBMCs). PBMCs from lean subjects were activated with a T cell-specific stimulus in the presence or absence of generally pro-inflammatory palmitate and/or non-inflammatory oleate. Palmitate decreased immune cell expression of NNT, NADPH, and anti-oxidant glutathione, but increased reactive oxygen and proinflammatory Th17 cytokines. Oleate had no effect on these outcomes. Genetic inhibition of NNT recapitulated the effects of palmitate. PBMCs from obese (BMI >30) compared to lean subjects had lower NNT and glutathione expression, and higher Th17 cytokine expression, none of which were changed by exogenous palmitate. Our data identify NNT as a palmitate-regulated rheostat of redox balance that regulates immune cell function in obesity and suggest that dietary or therapeutic strategies aimed at increasing NNT expression may restore redox balance to ameliorate obesity-associated inflammation.

## 1. Introduction

Metabolic diseases like obesity are characterized by mitochondrial dysfunction, redox imbalance, and chronic inflammation. However, the relationships amongst these physiological changes and how they promote metabolic dysfunction in obesity [1,2] is not well understood. Mitochondria generate cellular reactive oxygen species (ROS) mainly due to respiration-linked electron leakage that generates superoxide anion and other redox changes. These changes are kept in check by anti-oxidants under homeostatic conditions that are required for normal immune function [3]. Excess ROS, generated by over-exuberant mitochondria amidst deficiencies in anti-oxidants such as nicotinamide adenine dinucleotide phosphate (NADPH) and reduced glutathione (GSH), can trigger aberrant cellular signaling and other measures of damage that may upregulate pro-inflammatory cytokine expression [4] to pathogenic levels. Such mechanisms may support the unresolved inflammation that associates with obesity complications like type 2 diabetes (T2D) [5].

Nicotinamide nucleotide transhydrogenase (NNT) is an inner mitochondrial membrane protein that catalyzes the reduction of NADP^+^ by NADH and the conversion of NADH to NAD^+^ [6,7,8,9]. NNT has been implicated in redox balance and inflammation. Knockdown of NNT in human adrenocortical cells resulted in impaired redox potential, increased mitochondrial ROS and apoptosis [10]. Liver mitochondria from C57BL/6J mice, which lack NNT due to a spontaneous mutation, have major redox alterations including higher H_2_O_2_, spontaneous NADPH oxidation and lower GSH compared to a BL/6J sub-strain with a functional NNT gene [11]. Similarly, macrophages from C57BL/6J mice with an NNT transgene have lower ROS and less cytokine production than non-transgenic counterparts [12]. Whether ROS from specific organelle sources and/or antioxidant regulation by NNT simply parallels or directly causes pro-inflammatory cytokine production was not addressed by these studies. However, these data raise the possibility that obesity-associated mitochondrial dysfunction interpreted in the context of dyslipidemia perturb NNT, redox balance, and downstream outcomes like inflammation.

The objective of our study was to assess causal links amongst obesity-associated lipid shifts, NNT/ROS expression, and inflammation in peripheral blood mononuclear cells (PBMCs) from lean subjects to determine whether this pathway triggers the chronic inflammation that promotes obesity-associated metabolic decline. We used a combination of saturated and monounsaturated fatty acids, T cell-targeted stimuli, and genetic inhibition of NNT to show that palmitate decreases NNT and GSH to promote redox imbalance and thereby the Th17 cytokines that characterize inflammation in T2D [5]. PBMCs from obese subjects had lower expression of NNT and GSH compared to lean age-matched subjects that did not further change in response to palmitate ex vivo, consistent with the interpretation that this in vitro-induced pathway is active in people. Collectively these data indicate that dietary or therapeutic strategies aimed at increasing NNT expression may be beneficial for modulating mitochondrial redox balance to ameliorate obesity-associated inflammation.

## 2. Materials and Methods

### 2.1. Human Subjects Sample Collection

In accordance with the Declaration of Helsinki, informed consent for all human samples was obtained following Institutional Review Board-approved protocols at Boston University, University of Kentucky, and The Forsyth Institute (Ethical code numbers H27007, 44131, and 00000037, respectively). Peripheral blood was obtained from normoglycemic subjects who were lean (avg: 33 years; BMI 22.71 kg/m^2^) or obese (avg: 39.6 years; BMI 34.15 kg/m^2^), with additional characteristics shown in Table 1. Exclusion criteria were smoking, recent use of antibiotics or anti-inflammatory medications i.e., NSAIDs/steroids, recent (within two weeks) colds or flu, diabetes or diabetes medications, and any history of cancer, hyperglycemia, and auto-immune diseases.

### 2.2. Isolation of PBMCs and T Cells

Fifty milliliters of peripheral blood were collected into acid/citrate/dextrose containing tubes by venous puncture. PBMCs were purified by histopaque 1077 then frozen at −80 °C under controlled cooling conditions in a Mr. Frosty apparatus (Nalgene, Sigma Aldrich, St Louis, MO, USA). For multi-week storage, cells were moved to −170 °C following 1–7 days at −80 °C. PBMCs from the subjects were stimulated in vitro for 40 h with T cell-targeted αCD3/αCD28 Dynabeads (Thermo Fisher Scientific, 11132D, Waltham, MA, USA) at 2 μL Dynabeads per 100k cells. In some cultures, cells were co-treated with 400 µM palmitate (pal) (C16:0) coupled to fatty acid-free Bovine Serum Albumin (BSA) at a ratio of 2 mol palmitate to 1 mole BSA, or 400 µM oleate, or a combination of palmitate and oleate. These fatty acid concentrations mimic concentrations achievable in serum [13]. Control cells were treated with 1% BSA. The mitochondrial ROS scavenger MitoTempo (mito)(10 μM) or a general ROS scavenger N-acetyl cysteine (NAC) was added for the last 20 h of incubation (20 h post-stimulation) for some cultures. All treatments were done in RPMI media with 5 mM glucose (normoglycemic). Supernatants were collected and stored at −80 °C. Cells were assayed as outlined below.

### 2.3. Immunoblotting

Immunoblotting was used to quantify protein expression as we published [14,15]. The procedure was modified according to the cell type from which the proteins were extracted. Thirty μL of 1X cell lysis buffer (Cell Signaling Technology, Danvers, MA, USA) was added to 1 × 10^6^ cells and incubated on ice for 20 min. Cells were then centrifuged at 13,000 rpm for 20 min and supernatant was collected. A Bicinchoninic Assay (Thermo Fisher Scientific, Waltham, MA, USA) assessed protein concentration. Twenty μg protein was loaded in polyacrylamide gels and electrophoresis was performed at 100 V for 1 h. Transfer of protein to polyvinylidene difluoride (PVDF) membrane was performed at 45 V for 5 h. The membrane was blocked for 30 min at room temperature (RT) in blocking buffer containing 2% bovine serum albumin in TBST followed by overnight incubation at 4 °C in the respective primary antibodies. The membrane was washed 3X with 1X TBST and incubated with secondary antibodies for 2 h at RT, then imaged. Table 2 lists the antibodies used in this study. All antibodies were used at a dilution of 1:500 except β-actin which was used at 1:10,000. We quantified NNT, isocitrate dehydrogenase 2 (IDH2), malic enzyme 2 (ME2), glutamate cysteine ligase catalytic subunit (GCLC), heat shock protein 60 (Hsp60) and mitochondrial aconitase (m-aconitase) expression on western blots using Image studio lite (Licor, Lincoln, NE, USA) [16].

### 2.4. ROS Quantification

ROS was quantified using 2′,7′ –dichlorofluorescin diacetate (DCFDA) fluorescence (Sigma-Aldrich, D6883, St Louis, MO, USA) normalized to cell number [14,17]. Twenty-five mM glucose (Sigma Aldrich, D8270) and 50 µM tert-butyl hydrogen peroxide (TBHP) (Sigma Aldrich, 416665) were used as positive controls. The free radical scavenger Tempol (50 µM; Sigma Aldrich SML0737) was used to manipulate ROS generation and served as a control [14]. 

### 2.5. Mitochondrial Measures

Mitochondrial mass was quantified in PBMCs after stimulation +/− fatty acids using Mitotracker green fluorescence. PBMCs were incubated with αCD3/αCD28 ± 400 µM palmitate and/or oleate for 40 h then were washed twice with 1X PBS and incubated with 40 nM Mitotracker green (9074S, Cell Signaling Technology, Danvers, MA, USA) in 5 mM glucose-containing RPMI for 30 min. Mitotracker green fluorescence was assessed via fluorescence spectrophotometry (Biotek, Winooski, VT, USA) and the results were normalized to cell numbers.

### 2.6. Mitochondrial Membrane Potential

αCD3/αCD28-stimulated PBMCS ± palmitate and/or oleate were incubated with 400 nM tetramethylrhodamine, ethyl ester (TMRE), a cell-permeable positively-charged dye that accumulates in mitochondria with high membrane potential, for 30 min. The cells were assayed using microplate spectrophotometry (Biotek). Fifty µM FCCP was the positive control. 

### 2.7. Measurement of Cellular Antioxidants

Cellular GSH was measured using a colorimetric assay (BioVision, Milpitas, CA, USA) according to manufacturer’s protocol. Cellular NADPH was quantitated with a colorimetric assay according to manufacturer’s instructions (BioVision).

### 2.8. Measurement of Plasma Free Fatty Acid

Plasma free fatty acids were quantitated using EnzyChrome free fatty acid assay kit (BioAssay Systems, EFFA 100, Hayward, CA, USA) according to manufacturer’s instructions.

### 2.9. Cytokine Analysis

The Milliplex Th17 cytokine multiplex kit (Millipore, Burlington, MA, USA) was used to assess protein accumulation in all supernatants in technical duplicate on a Luminex FlexMAP 3D instrument (Luminex, Austin, TX, USA). Alternatively, we quantified IL-17A/F and IL-23 using the Legend Max human ELISA kit (Biolegend, San Diego, CA, USA), performed according to manufacturer’s instructions. The IL-17A/F ELISA does not distinguish between the two isotypes, which heterodimerize to mediate a large portion of “IL-17” activity [18]. 

### 2.10. Small Interference RNA

Small interference RNA (siRNA)-mediated knockdown of NNT was performed using Accell siRNA (Dharmacon, Lafayette, CO, USA) following manufacturer’s guidelines. Scrambled sequences were used as controls. The siRNA (1 µM) was diluted in Accell siRNA delivery medium and added to PBMCs with 2% FBS and 300 IU of IL-2. Cells were incubated ~40 h prior to assessing knockdown efficiency via immunoblotting and subsequent analysis. 

### 2.11. Statistical Analyses

Data are presented as mean ± standard error of the mean (SEM). For comparison among three or more groups a one-way ANOVA and a Tukey’s post hoc test was used to identify differences. Unpaired 2-tailed *t*-tests compared means of two values. Significance was accepted when *p* < 0.05. 

## 3. Results

### 3.1. Palmitate Decreased PBMC Membrane Potential

**Figure 1:**
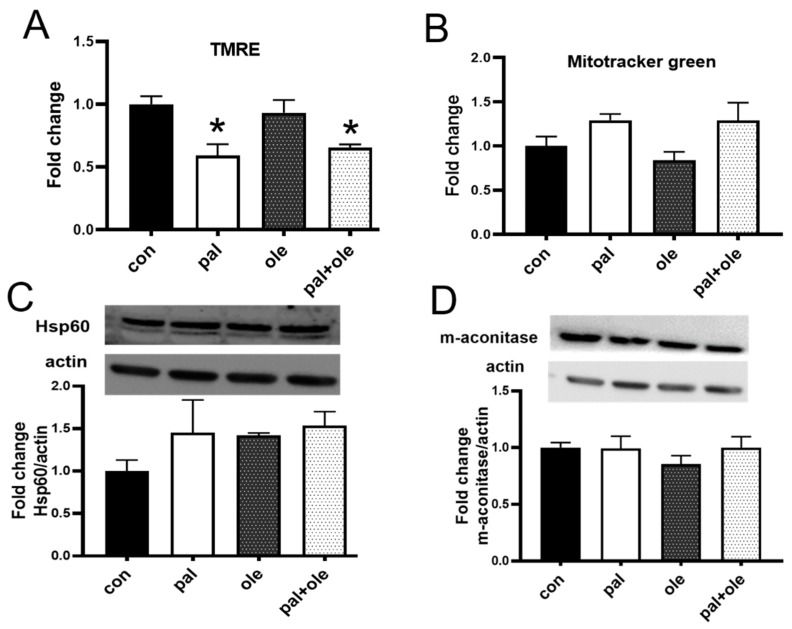
Palmitate decreased peripheral blood mononuclear cells (PBMC) membrane potential without altering mitochondrial mass: PBMCs isolated from normoglycemic, lean subjects (ave: 33 years; BMI 22.71 kg/m^2^) were treated with 400 μM palmitate (pal) or oleate (ole) or a mixture of palmitate and oleate (pal + ole) prior to measuring (**A**) Mitochondrial membrane potential with tetramethylrhodamine ethyl ester (TMRE); (**B**) Mitochondrial mass with mitotracker green fluorescence, or mitochondrial inner membrane proteins (**C**) Hsp60 and (**D**) m-aconitase. N = 5 each treatment. Each N represents cells from one subject. * *p* < 0.05 vs. control (con).

Fatty acid oxidation by T cell mitochondria regulates T cell function [19], but the impact of free fatty acids (FFAs) on mitochondrial membrane potential and mass, as preliminary indicators of T cell function, is untested. We activated PBMCs from lean subjects with T cell-specific αCD3/αCD28 in the presence of 400 μM palmitate or oleate alone or in combination, then quantified membrane potential and mitochondrial content. Palmitate alone or in combination with oleate decreased membrane potential, but oleate alone had no effect, as measured by TMRE fluorescence (Figure 1A). Mitochondrial content was similar amongst treatments, as measured by Mitotracker green fluorescence and expression of the inner mitochondrial proteins Hsp60 and m-aconitase on Western blots (Figure 1B–D). We conclude that palmitate dissipates PBMC mitochondrial membrane potential without changing mitochondrial mass, and that oleate cannot restore baseline membrane potential in the presence of palmitate.

### 3.2. Palmitate Altered the Cellular Redox Balance

**Figure 2:**
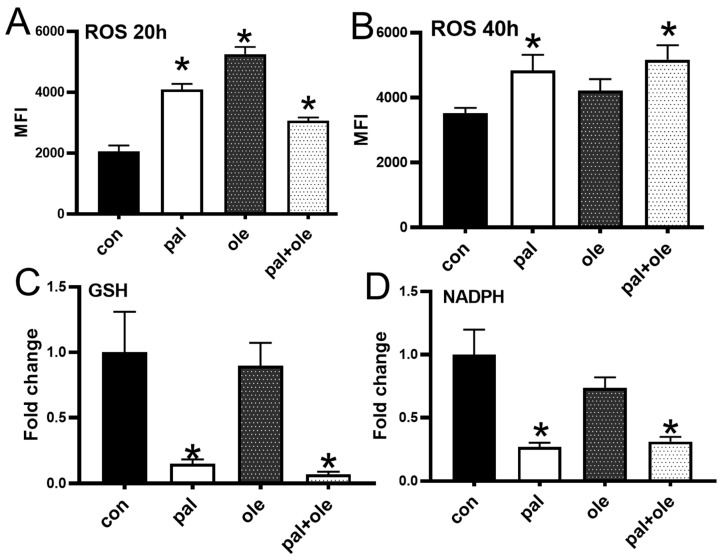
Palmitate treatment of PBMCs altered cellular redox balance: Cellular reactive oxygen species (ROS) at (**A**) 20 h post activation or (**B**) 40 h post activation in the presence or absence of palmitate (pal) and oleate (ole) per Figure 1. Y axis is mean fluorescence intensity (MFI), an approximation of the number of molecules of ROS in the cells. (**C**) reduced glutathione (GSH), and (**D**) NADPH were measured at 40 h post-stimulation +/− pal/ole. N = 6–8 per treatment. Each N represents cells from one subject. * *p* < 0.05 vs. control (con).

The observation that palmitate lowered membrane potential in PBMCs suggested that palmitate may change redox status to lower ROS generation by these cells. To the contrary, palmitate alone or in combination with oleate increased ROS generation compared to cultures lacking exogenous FFAs, although oleate effects were time-dependent (Figure 2A,B). Glutathione (GSH), a dominant cellular antioxidant/redox regulator, and NADPH were dramatically decreased by palmitate alone or in combination with oleate (Figure 2C,D). NADP concentrations were highly variable, thus interpretation was tenuous. We conclude palmitate significantly disrupts redox homeostasis of immune cells from lean subjects, while early effects of oleate on ROS may be moderated over time.

### 3.3. Palmitate Lowered the Expression of Nicotinamide Nucleotide Transhydrogenase

**Figure 3:**
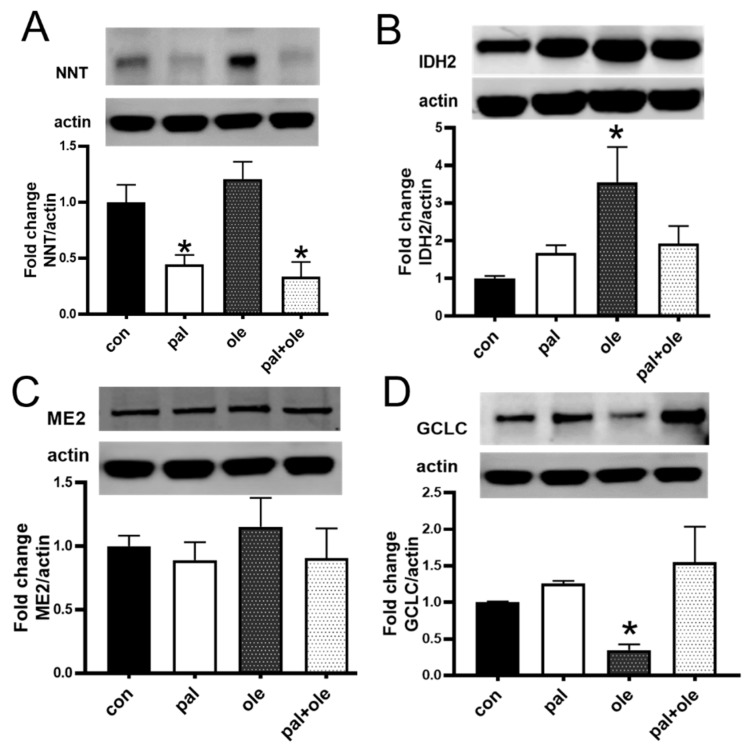
Palmitate lowers nicotinamide nucleotide transhydrogenase (NNT) protein expression: Cellular (**A**) NNT, (**B**) isocitrate dehydrogenase 2 (IDH2), (**C**) malic enzyme 2 (ME2), or (**D**) the glutamate cysteine ligase catalytic subunit were measured relative to the β actin loading control on Western blots to assess the redox regulators in PBMCs stimulated with αCD2/αCD28 in the presence or absence of palmitate (pal) and oleate (ole) per Figure 1. In panel A, N = 7–8. For Panels B,C&D, N = 5. Each N represents cells from one subject. * *p* < 0.05 vs. control (con).

GSH generation requires NADPH, which is regulated by several mitochondrial proteins including NNT, isocitrate dehydrogenase (IDH2), malic enzyme (ME2) and glutamate cysteine ligase (GCL). We quantified the effect of palmitate alone or in combination with oleate on these four indirect regulators of redox balance. NNT was significantly decreased by palmitate, but not oleate, in αCD3/αCD28-stimulated PBMCs (Figure 3A). IDH2, ME2 and GCLC were unchanged by palmitate (Figure 3B–D), but oleate significantly increased IDH2 and decreased GCLC protein expression (Figure 3B–D). These findings support the conclusion that palmitate-mediated decreases in NNT, but not IDH2, ME2 or GCLC, are associated with lower mitochondrial membrane potential and redox disruption in PBMCs. Furthermore, oleate-mediated changes in other potential GSH regulators did not associate with ROS differences, indicating oleate failed to alter multiple mitochondrial proteins critical for redox homeostasis.

### 3.4. Palmitate Increased Proinflammatory Cytokine Production by PBMCs

**Figure 4:**
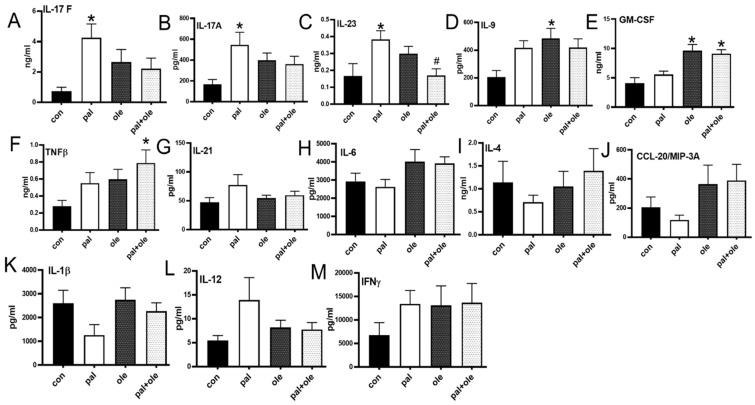
Palmitate promotes production of characteristic Th17 cytokines. Cytokine production from cells stimulated in the presence or absence of palmitate (pal) and oleate (ole) per Figure 1 and assessed via multiplex. The concentrations of other cytokines assessed by the multiplex assay (IL-10, IL-13, IL-15, IL-22, IL-33, IL-2, IL-5, IL-17E, IL-27, IL-31, TNFα, TNF-β, IL-28A) remained unchanged after the different treatments (not shown). N = 9–10 in each treatment. Each N represents cells from one subject. * *p* < 0.05 vs. control (con). Interleukin (IL), Tumor Necrosis Factor α (TNFα), Tumor Necrosis Factor β (TNFβ), Interferon γ(IFNγ), Chemokine (C-C motif) ligand 20 (CCL20)/ Macrophage Inflammatory Protein-3 (CCL20/MIP3A), Granulocyte Macrophage Colony Stimulating Factor (GM-CSF).

T cell cytokines promote insulin resistance in animal models of obesity [20,21], while Th1 and Th17 cytokines mathematically predict glycemic control and type 2 diabetes, respectively, in people [5]. To identify links between palmitate-induced redox/mitochondrial changes and obesity-associated inflammation, we stimulated PBMCs with αCD3/αCD28 in the presence of palmitate and quantified cytokines. Palmitate increased production of multiple (but not all) Th17-associated cytokines (IL-17A, -17F, and -23, but not IL-21) with insignificant/variable effects on Th1 (IL-6, IL-12, IFNγ) and Th2 (IL-4) cytokines (Figure 4). Oleate treatment had no effect on classically defined Th17 cytokines, but increased GMCSF and IL-9 (Figure 4 D,E), which have multiple cellular origins [22,23,24] and pleotropic effects on inflammation. Unstimulated PBMCs produced low/unmeasurable amounts of all cytokines measured (data not shown). We conclude that palmitate-associated changes in mitochondrial function and redox balance parallel Th17 cytokine activation in immune cells from lean subjects.

### 3.5. NNT Protein Knockdown Recapitulated Palmitate-Induced Changes in PBMCs

**Figure 5:**
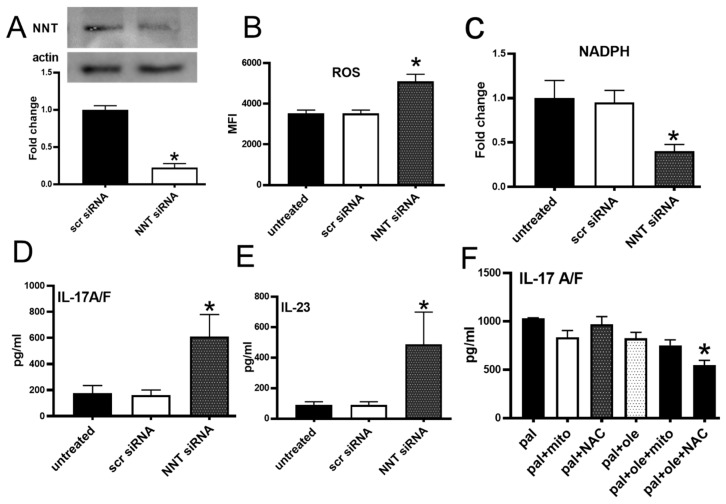
NNT knockdown promotes ROS and Th17 cytokine production: (**A**) NNT protein measured following knockdown with scrambled (scr) or NNT-targeted siRNA. Amounts of cellular (**B**) ROS (DCFDA), (**C**) NADPH (measured using a commercial kit), (**D**) IL-17A/F (in ELISA) and (**E**) IL-23 (in ELISA). (**F**) IL-17A/F quantified after stimulation in the presence of the indicated additives: palmitate (pal), oleate (ole), MitoTempo (mito) (10 μM) or N-acetyl cysteine (NAC, 20 μM). A,B,C,F N = 5, D&E N = 7–8 in each treatment. Each N represents cells from one subject. * *p* < 0.05 vs scr siRNA treated cells (A–E) or pal in F.

To establish cause/effect relationships between palmitate-mediated decrease in NNT expression and the Th17 inflammation that characterizes obesity-associated type 2 diabetes [5], we treated PBMCs from lean subjects with NNT-specific siRNA and analyzed redox and cytokine outcomes. We confirmed NNT siRNA decreased NNT protein ~75% relative to a scrambled siRNA control (Figure 5A). Analysis of ROS and NADPH in the NNT knock-down showed the predicted changes (Figure 5B,C). Both IL-17A/F and IL-23 expression were significantly higher in NNT knockdown cells compared to control cells (Figure 5D,E). We conclude that experimental NNT knockdown recapitulates palmitate-downstream redox imbalance and Th17 cytokine up-regulation.

Th17 cytokines require 32–40 h for detectable expression, raising the possibility that ROS from either mitochondrial and/or non-mitochondrial sources may be required at multiple points along this relatively long timeline for NNT-mediated inflammation. To test the importance of ROS in maintenance of the events that culminate in Th17 cytokine production many hours later, we added MitoTempo(mito) or a general ROS scavenger N-acetyl cysteine (NAC) at 20 h post-stimulation, and quantified cytokines by ELISA at 40 h. MitoTempo failed to impact IL-17A/F production under palmitate +/ oleate co-treatment (Figure 5F), supporting the interpretation that palmitate/NNT-triggered mitochondrial ROS is not required for maintenance of cytokine production. Although NAC significantly blunted IL-17A/F production, interpretation is confounded by the ability of NAC to activate the proteasome and thus affect multiple NF-κB regulated inflammatory genes [25]. Addition of MitoTempo at time zero significantly compromised cell viability, making interpretations of the role of early mitochondrial ROS on the palmitate-NNT-Th17 pathway uncertain. We conclude that an initial burst of mitochondrial ROS in the first 20 h post-stimulation, perhaps in combination with the ROS-induced ROS generation likely induced at later time points, is sufficient to support Th17 cytokine production in response to palmitate.

### 3.6. PBMCs from Subjects with Obesity Mimicked Characteristics of Redox Imbalance Caused by Palmitate

**Figure 6:**
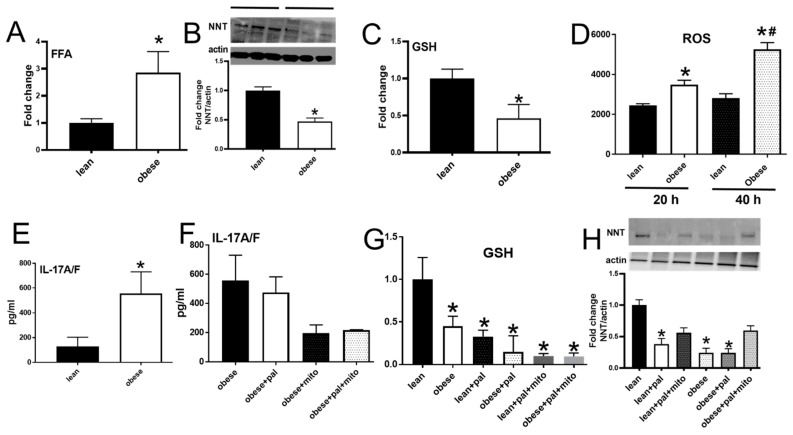
NNT expression was blunted in PBMCs from obese non-diabetic subjects. Circulatory free fatty acid levels were assessed in the plasma of lean and obese subjects (**A**) PBMCS from lean or obese subjects (Table 1) were analyzed for (**B**) NNT on Western blots (**C**) glutathione (**D**)ROS production 20 h and 40 h post activation and (**E**) IL-17A/F production. The production of (**F**) IL-17A/F (**G**) GSH and (**H**) NNT expression was assessed after palmitate +/− the mitochondrial free radical scavenger MMitoTempo (mito). A N = 6, B&D N = 5, C,E,F,G,H N = 7–8. Each N represents one subject. * *p* < 0.05 vs lean. # *p* < 0.05 vs. obese.

The known increase in circulating FFAs in obesity [26,27] was recapitulated in our obese cohort (Figure 6A). These data raise the question of whether the palmitate-sensitive redox regulators revealed by our analyses of cells from lean people is mirrored in cells from the obese cohort. Both NNT and GSH expression were lower in αCD3/αCD28-stimulated PBMCs from obese compared to lean subjects (Figure 6B,C), and ROS was higher at both time points tested (Figure 6D), as was IL-17A/F (Figure 6E). Exogenous palmitate did not change any measures of redox imbalance or inflammation in cells from obese subjects (Figure 6F–H), suggesting redundant regulatory pathways. MitoTempo had no impact on IL-17A/F, GSH or NNT in cells from obese subjects, indicating that non-mitochondrial sources of ROS added a layer of regulation absent in palmitate-treated cells from lean subjects. These findings support the interpretation that higher circulating palmitate in obese subjects supports redox imbalance that stems from both mitochondrial and non-mitochondrial sources and culminates in increased production of T2D-associated Th17 cytokines [5].

## 4. Discussion

How obesity in people triggers the chronic inflammation that promotes the transition from obese/normoglycemic to obese/metabolically compromised remains an important outstanding question with significant clinical impact. Identifying nutrient-sensitive mechanisms that activate inflammation alone or in the presence of generally non-inflammatory nutrients (oleate) will aid experimental design for clinical trials on nutrients as sustainable mediators of the events that fuel obesity co-morbidities. Our work links multiple previous studies on FFAs, mitochondrial (dys)function and inflammation to support the conclusion that saturated FFAs like palmitate down-regulate the mitochondrial protein NNT to trigger a cascade of events leading to the Th17 inflammation that associates with T2D. Our data do not eliminate the possibility of NNT-independent pathways despite elimination of bioenergetically complex regulators like ME2 [28,29], but instead, define a previously unappreciated pathway from a dietary nutrient to inflammation.

**Figure 7:**
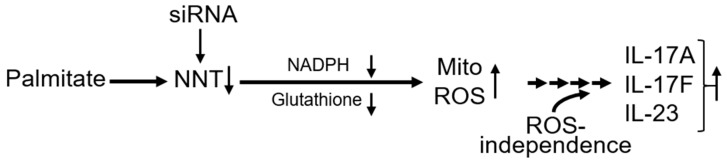
Schematic description of the findings. The saturated fatty acid palmitate blunts the expression of the mitochondrial redox regulator NNT. This leads to decreased cellular NADPH and glutathione anti-oxidant, resulting in a redox imbalance, more mitochondrial ROS, and higher concentrations of proinflammatory Th17 cytokines. These events become mitochondrial ROS-independent following 20 h of stimulation, and partially recapitulate the T2D-associated Th17 cytokine profile. Knock-down of NNT by siRNA recapitulates the effects of palmitate.

Several lines of evidence are consistent with the model generated from our results (Figure 7). In addition to outcomes from C57BL/6 mice that have a breeding-generated NNT mutation and cellular redox imbalance compared to NNT-expressing mice [11,12], NNT also appears to regulate redox in people. Demonstrations that NNT regulates redox balance in clinically relevant settings include an NNT mutation in patients with familial glucocorticoid deficiency [10], who develop organ pathologies related to impaired anti-oxidant defense mechanisms. Altered redox following NNT silencing in human adrenocortical or PC12 cells confirmed this connection ex vivo [10,30]. NNT may also protect MnSOD-deficient mitochondria [31], further linking NNT to redox balance.

NNT has also been identified as a regulator of metabolic health in multiple studies. Quantitative trait locus mapping highlighted NNT as important for glycemic control and insulin secretion in C57BL/6 mice, although the investigators assumed NNT function was mainly in the pancreatic beta cells [32,33]. Comparison of C57BL/6 mice +/- functional NNT showed NNT protected against glucose intolerance upon high fat diet feeding, though the underlying cause of greater weight gain (which generally challenges euglycemia) in the absence of NNT was not investigated [34]. NNT mRNA expression was assessed in visceral and subcutaneous adipose tissues of 221 human subjects of wide range of BMI, insulin sensitivity and glucose tolerance. The study concluded that, in contrast to findings herein and to published work in mice, NNT mRNA expression was higher in the visceral fat of obese subjects and positively correlated with BMI, body weight, percent body fat, waist to hip circumference and fasting plasma insulin [35]. The authors raise the possibility that higher NNT resulted in feed forward loops that culminate in beta cell “burn out” amongst other explanations for this unexpected finding. Another study reported that NNT and GSH mRNA, among other indicators of mitochondrial function, increase in subcutaneous and visceral adipose tissue 12 months after sleeve gastrectomy along with a decrease in adipokine mRNAs (a measure of inflammation) [36]. It is important to note that the impact of bariatric surgery on inflammation is variable across studies [37] The results from these studies disagree indicating that further work is needed to determine whether technical differences (mRNA vs. protein quantification), cell type differences (total visceral adipose tissue vs. circulating immune cells), or species differences (mouse vs. human) explain the apparent discrepancies amongst these findings. Newly appreciated links from FFA excess to NNT/redox imbalance terminating in obesity-associated Th17 inflammation may more broadly represent a regulatory pathway that drives functions of other cell types in obesity through similar mitochondrial machinery. NNT-targeted interventions may therefore normalize multiple cellular functions that perpetuate obesity-associated complications.

The T2D-associated Th17 profile was derived from a comparison of obese subjects with and without T2D [5]. It is difficult to say whether partial recapitulation of this profile (IL-17A, -17F, -23) by palmitate treatment of cells from lean subjects herein is more modeling the obese/normoglycemic status tested here, or the obese/T2D status that was tested by our profiling work. Partial recapitulation of the profile by palmitate could mimic the physiology somewhere along the continuum of progression from obesity to obesity-associated T2D, a speculation that would require substantial additional investigation.

## Figures and Tables

**Table 1:** Description of research subjects.

	Lean	Obese
Total N	12	8
Age, years [mean (range)]	33 (28–45)	39.6 (30–47)
A1c, % [mean (range)]	5.1 (4.5–5.6)	5.3 (5–5.5)
BMI, kg m^−2^ [mean (range)] *	22.71 (21.1–24.4.)	33.94 (30.4–40.8)
Females [N (%)]	7 (58.33%)	4 (50%)
Males [N (%)]	5 (41.66%)	4 (50%)

* *p* < 0.05 compared to lean.

**Table 2:** Antibodies used in this study.

Protein	Catalog #	Company
NNT	ab110352	Abcam
m-aconitase	ARP40390_P050	Aviva biologicals
Hsp60	12165S	Cell signaling technology
GCLC	ARP54577_P050	Aviva biologicals
IDH2	ab131263	Abcam
B-Actin	3700	Cell signaling technology
Malic enzyme	12399S	Cell signaling technology

All antibodies were used at a dilution of 1:500 except β-actin (1:10,000).
